# How *Escherichia coli* lands and forms cell clusters on a surface: a new role of surface topography

**DOI:** 10.1038/srep29516

**Published:** 2016-07-14

**Authors:** Huan Gu, Aaron Chen, Xinran Song, Megan E. Brasch, James H. Henderson, Dacheng Ren

**Affiliations:** 1 Department of Biomedical and Chemical Engineering, Syracuse University, Syracuse, NY 13244, USA; 2 Syracuse Biomaterials Institute, Syracuse University, Syracuse, NY 13244, USA; 3 Department of Civil and Environmental Engineering, Syracuse University, Syracuse, NY 13244, USA; 4 Department of Biology, Syracuse University, Syracuse, NY 13244, United States

## Abstract

Bacterial response to surface topography during biofilm formation was studied using 5 μm tall line patterns of poly (dimethylsiloxane) (PDMS). *Escherichia coli* cells attached on top of protruding line patterns were found to align more perpendicularly to the orientation of line patterns when the pattern narrowed. Consistently, cell cluster formation per unit area on 5 μm wide line patterns was reduced by 14-fold compared to flat PDMS. Contrasting the reduced colony formation, cells attached on narrow patterns were longer and had higher transcriptional activities, suggesting that such unfavorable topography may present a stress to attached cells. Results of mutant studies indicate that flagellar motility is involved in the observed preference in cell orientation on narrow patterns, which was corroborated by the changes in cell rotation pattern before settling on different surface topographies. These findings led to a set of new design principles for creating antifouling topographies, which was validated using 10 μm tall hexagonal patterns.

The vast majority of bacteria on earth live in biofilms, which are sessile structures of matrix-encased microorganisms found ubiquitously on both biotic and abiotic surfaces^1^,^2^. Cell adhesion is the first step in biofilm formation and is affected by many surface properties such as surface topography, chemistry, charge, and stiffness^3^,^4^,^5^. Thus, antifouling strategies based on surface and material engineering have been extensively explored in the past decade due to the growing awareness of biofilm associated challenges in medicine and industry^6^,^7^. Among the biofilm control strategies, engineering surface topography has attracted special attention recently due to its potential to prevent bacterial adhesion and subsequent biofilm formation without using antimicrobial agents^8^,^9^,^10^,^11^,^12^,^13^,^14^,^15^,^16^,^17^,^18^,^19^,^20^,^21^,^22^.

By using nano and submcron scale (size that is comparable or smaller than that of a single bacterial cell) protruding^13^,^14^,^16^,^17^,^18^,^19^,^20^,^21^,^22^ or recessive^8^,^9^,^11^,^16^ features, surface topographies were found to change total surface area^19^ and surface wetness^19^ and thus either positively or negatively influence biofilm formation by affecting cellular activities^9^, expression of outer membrane appendages^8^,^9^, or the function of bacterial flagella^19^,^23^. Although it is well recognized that surface topography affects bacterial adhesion and biofilm formation, the influence of surface topography on the physiology of attached cells is still poorly understood, hindering the rational design of antifouling surface topographies.

Recently, we reported that the attachment of *E. coli* cells on top of 10 μm tall protruding square shaped PDMS patterns is significant only if the patterns are 20 μm × 20 μm or bigger for face-up patterns and 40 μm × 40 μm or bigger for face-down patterns^24^. The existence of these threshold dimensions suggests that bacterial cells may actively explore surface topography to decide the switch between planktonic growth and biofilm formation on top of topographic features^24^. Since these threshold dimensions are much bigger than the average size of *E. coli* cells (~2 μm in length and 0.25–1.0 μm in diameter^25^), cell-cell interactions may also be essential to biofilm formation on these surfaces. One factor that is known to be important to the cell-cell interaction and the following steps of biofilm formation is the orientation of attached cells due to its critical role in the physical interactions between neighboring cells and some critical cellular processes such as cell division and signaling^20^,^26^,^27^. However, how bacteria adjust cell orientation in response to micron scale surface topographies has not been studied. This motivated us to investigate the influence of micron scale topographies (dimensions larger than the size of single bacterial cells) on the orientation and morphology of attached cells and the subsequent biofilm formation using PDMS as a model material. We chose PDMS because it is a commonly used biomaterial^13^,^16^,^19^,^22^ and allows us to compare with previous studies using this material^19^,^22^,^24^.

## Results

### *E. coli* cells attached on top of narrow line patterns exhibited preference in cell orientation

**Figure 1:**
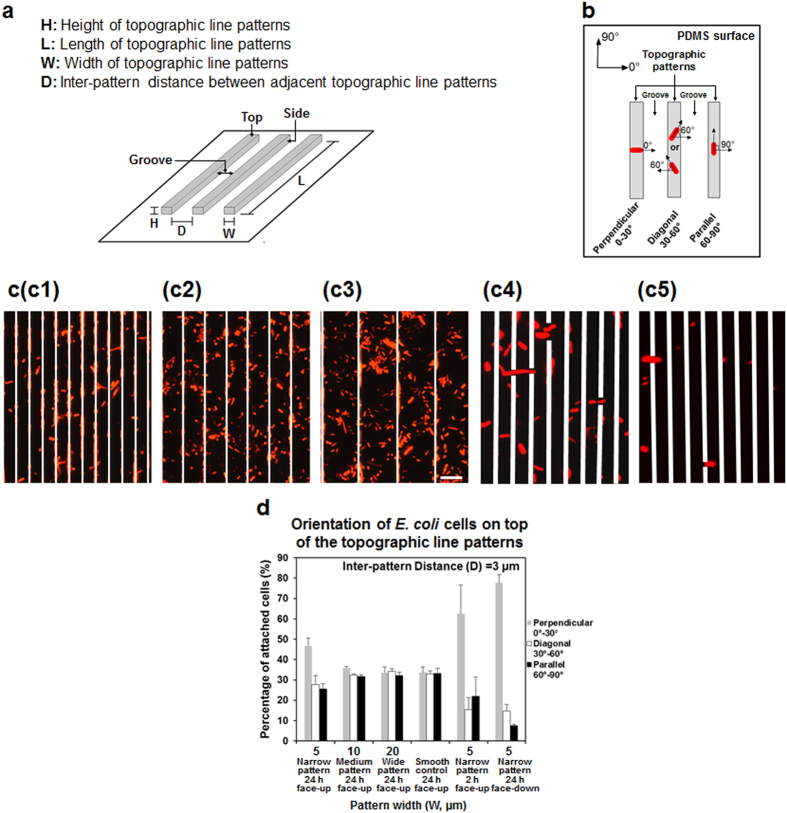
Orientation of the WT *E. coli* cells attached on top of PDMS line patterns at 24 h after inoculation. (**a**) Schematic description of PDMS line patterns used in this study. Pattern Length (L) and Height (H) were fixed at 4 mm and 5 μm, respectively, while pattern width (W) and inter-pattern distance (D) were varied. (**b**) Definition of cell orientation. Cells attached to both the top and side of line patterns and in the grooves, but only the cells attached on top of line patterns were compared for cell orientation. (**c**) Representative fluorescence images of WT *E. coli* cells attached on top of 5 μm (c1), 10 μm (c2), or 20 μm (c3) wide face-up line patterns after 24 h biofilm growth, 5 μm (c4) wide face-up line patterns at 2 h after initial attachment, and 5 μm (c5) wide face-down line patterns after 24 h biofilm growth (Bar = 10 μm). Cells were also found attached on the side of protruding line patterns and in the grooves between topographic line patterns. These cells are covered with white lines to more clearly show the cells on top of the line patterns (the focus of this study). (**d**) Distribution of cell orientation on top of face-up narrow (5 μm wide), medium (10 μm wide), or wide (20 μm wide) PDMS line patterns after 24 h biofilm growth, face-up narrow patterns at 2 h after initial attachment, and face-down narrow line patterns after 24 h biofilm growth with 3 μm inter-pattern distance (mean ± standard error shown; at least three biological replicates were tested for each sample). Smooth PDMS was included as control.

Previous studies have shown that bacteria cells prefer to attach and form cell clusters in the grooves between protruding features^9^,^16^,^18^,^20^,^24^,^28^,^29^,^30^. Cells that settle between topographic patterns prefer to align in parallel to the orientation of topographic features (e.g., nanoposts, squares, or lines) to maximize the surface of contact^9^,^18^,^20^,^30^. Hence, we hypothesized that *E. coli* cells attached on top of narrow line patterns may also align along the line orientation to maximize the contact with the surface and allow cell growth. To test this hypothesis, we grew 24 h *E. coli* RP437/pRSH103^31^ (henceforth WT *E. coli*) biofilms on smooth PDMS surfaces and PDMS modified with 5 μm tall line patterns with varying width (W) [Narrow (W = 5 μm), Medium (W = 10 μm), and Wide (W = 20 μm); Fig. 1a] in static Lysogeny Broth (LB) at 37 °C. All the biofilm formation experiments in this study were conducted on face-up PDMS surfaces (including modified PDMS surfaces and smooth controls), if not noted otherwise. The inter-pattern distance (D) was varied to be D = 3, 5, 10, or 20 μm (Fig. 1a). Specifically, we focused on the cells attached on top of protruding lines, rather than those attached on the side of line patterns or in the grooves between protruding lines (Supplementary Fig. S1). Over 10,000 WT *E. coli* cells were imaged and analyzed (from at least 3 biological replicates with 6 positions randomly selected and imaged for each sample). Cell orientation was defined as perpendicular (0–30°), diagonal (30–60°), or parallel (60–90°) with respect to the orientation of the lines (Fig. 1b).

It was found that pattern width has profound effects on cell orientation on top of the line patterns (Fig. 1c,d), while inter-pattern distance (width of groove) showed no influence (Supplementary Fig. S2). However, unlike what we hypothesized, cell orientation on top of narrow line patterns (5 μm wide) was more skewed towards a perpendicular orientation with respect to the direction of the line patterns (0–30° on narrow patterns) (*p* < 0.0001, two way ANOVA followed by Tukey test) instead of along the line direction. As shown in Fig. 1d and Supplementary Fig. S2, on patterns with 5 μm width and 3 μm inter-pattern distance, more cells (46.7 ± 6.8% of the total cells) oriented between 0–30° (perpendicular to line patterns), followed by those between 30–60° (27.8 ± 7.7%) and 60–90° (25.6 ± 4.5%). On the top of medium width patterns (W = 10 μm), the cell orientation were found to be more uniformly distributed than on the narrow patterns, yet exhibited a slight, albeit consistent, skew towards a perpendicular orientation (Fig. 1d, *p* < 0.0001, two way ANOVA followed by Tukey test). The wide patterns showed a near-uniform distribution of attachment angles (Fig. 1d) (*p* > 0.05, two way ANOVA followed by Tukey test), which was also found on smooth surfaces.

Previously, Diaz *et al.*^18^ reported that that *Pseudomonas fluorescens* cells on top of submicron line patterns (840 nm wide, 120 nm deep, and 320 nm inter-pattern distance) on copper surfaces aligned in 0–45° (the orientation that is perpendicular to the orientation of line patterns was set as 0°) to the line patterns. The bias in orientation was attributed to the need to maximize cell-surface contact. The line patterns used in the present study are much wider (5–20 μm); thus, contact area is not a critical factor for the orientation profile observed here.

### Preference in cell orientation involves cellular activities

To further understand if the aforementioned preference in cell orientation in 24 h biofilms was established upon initial attachment or during biofilm growth, the orientation of the WT *E.coli* cells on top of 5 μm wide line patterns after 2 h of initial attachment was also analyzed. The result showed that these cells also aligned more perpendicularly to the direction of 5 μm wide line patterns (Fig. 1d and Supplementary Fig. S3; *p* < 0.0001, two way ANOVA followed by Tukey test). In fact, the percentage of cells that oriented perpendicularly after 2 h of inoculation was even higher than that in 24 h biofilms. For example, 62.5 ± 24.4% cells (compared to 46.7 ± 6.8% cells in 24 h biofilms) attached perpendicularly to the 5 μm wide line patterns with 3 μm inter-pattern distance (Fig. 1d and Supplementary Fig. S3). The cell orientation on flat surface (as control) after 2 h of adhesion was also random as observed after 24 h. Collectively, these results suggest that the perpendicular orientation is strongly favorable for initial attachment, which was retained in 24 h biofilms as described above.

Both physical factors (Brownian motion, gravity, electrostatic interactions, hydrodynamics, and van der Waals forces)^32^,^33^ and bacterial activities (bacterial motility^34^, production of exopolysaccharides^29^, and functions of outer membrane structures)^19^,^35^,^36^ can affect the initial cell attachment to a surface during biofilm formation. Similar results of cell orientation was also observed when we repeated 24 h biofilm formation on face-down PDMS surfaces with narrow patterns (Fig. 1d and Supplementary Fig. S4), suggesting that the preference in cell orientation was not due to gravity driven cell settlement. To understand if any cellular activities are involved in the preference of cell orientation on top of narrow line patterns, the attachment on top of 5 μm wide face-up line patterns was repeated with the cells pretreated with 10 μg/mL chloramphenicol (to inhibit protein synthesis)^37^ for 1 h before inoculation. As shown in Supplementary Fig. S5, the orientation of these pretreated WT *E. coli* cells after 24 h incubation was more skewed toward diagonal (30–60°) orientation rather than perpendicular (0–30°) orientation observed for untreated cells (Fig. 1d; *p* < 0.0001, two way ANOVA followed by Tukey test). Because chloramphenicol represses protein synthesis and thus inhibits major cellular functions, we speculate that the observed preference in cell orientation on top of narrow line patterns may be not simply due to physical factors, but involve cellular activities.

### Mutation of key genes altered cell orientation on line patterns

To further understand what bacterial activities are involved in the observed preference in cell orientation, the 24 h biofilm formation on PDMS surfaces modified with 5 μm wide line patterns was repeated with four isogenic mutants of the WT *E. coli*: *fliC* (RHG01), *motB* (RP3087), *fimA* (RHG02), and *luxS* (KX1485). These mutants were selected because the corresponding genes are important to bacterial adhesion and biofilm formation^19^,^35^,^36^,^38^,^39^,^40^,^41^. The *fliC* gene encodes flagellin, which is the major component of flagella^38^. The *motB* gene encodes a stator protein MotB, which plays an important role in the control of flagellar rotation and the transmission of mechanical signals^35^. The *fimA* gene encodes the subunits of fimbriae^40^, which are bacterial appendages involved in adhesion and movement on a solid surface^33^,^37^,^39^. To understand if cell-cell signaling is important, we also included a mutant of *luxS* gene, which is essential for the synthesis of quorum sensing signal AI-2^41^.

**Figure 2:**
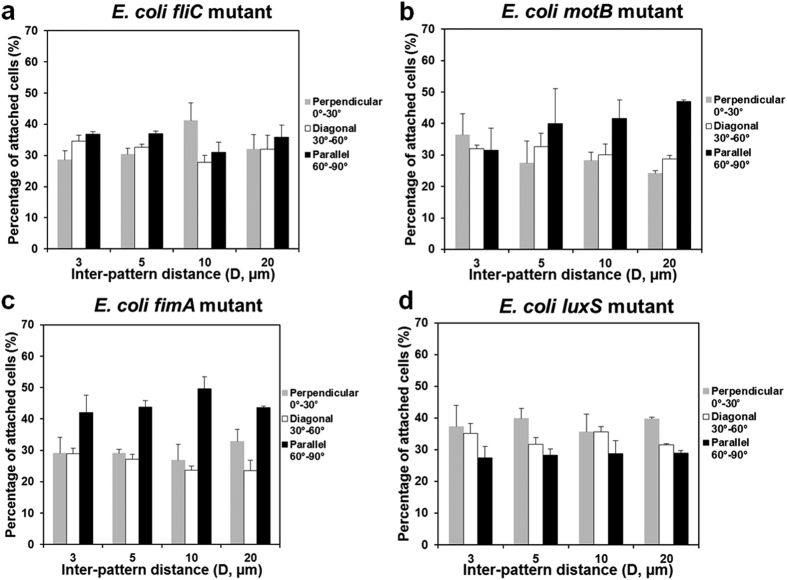
Orientation of isogenic mutant cells on top of 5 μm wide line patterns (mean ± standard error shown; at least three biological replicates were tested for each sample). (**a**) *fliC* mutant (*E. coli* RHG01/pRSH103), (**b**) *motB* mutant (*E. coli* RP3087/pRSH103), (**c**) *fimA* mutant (*E. coli* RHG02/pRSH103), (**d**) *luxS* mutant (*E. coli* KX1485/pRSH103).

Mutation of *fliC*, *motB*, or *fimA* abolished the preference in perpendicular orientation on top of 5 μm wide patterns exhibited by the WT *E. coli* strain (Fig. 2). For example, the orientation of *fimA* mutant is actually skewed toward parallel orientation (Fig. 2c). To confirm that the results of *motB*, *fliC*, and *fimA* mutants were not due to any polar effects, we complemented the mutants with plasmids pRHG03, pRHG04, and pRHG05 carrying *motB*, *fliC*, and *fimA* genes, respectively, all under the control of a *lac* promoter. As shown in Supplementary Fig. S6, the defects of all three mutants were rescued by complementation. For example, cell orientation became random on patterns with 5 μm width and 3 μm inter-pattern distance after the deletion of *fliC* gene. The preference in cell orientation was restored after the complementation of *fliC* gene; e.g., more cells (48.8 ± 4.7%) oriented between 0–30° (perpendicular to line patterns), followed by those between 30–60° (24.8 ± 1.1%) and 60–90° (26.4 ± 3.7%). Thus, all these three genes appeared to be important to the preference in cell orientation on top of narrow line patterns. In comparison, mutation of the gene *luxS* only partially reduced the difference in cell orientation, but did not fully abolish it (Fig. 2d; *r* < −0.5 for all patterns studied, Pearson correlation analysis; *p* < 0.0005 for inter-pattern distance of 5 and 20 μm and *p* > 0.05 for inter-pattern distance of 3 and 10 μm). Hence, *luxS* gene is not essential to the preference in orientation.

### Bacterial flagella are involved in the arrangement of cell orientation on top of line patterns

**Figure 3:**
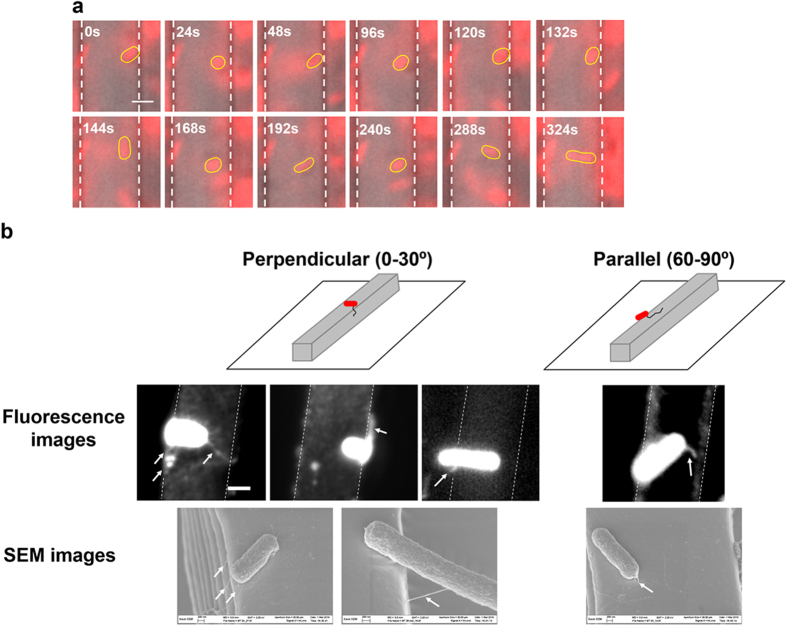
*E. coli* attachment on top of 5 μm wide line patterns. (**a**) A series of time-lapse images of a representative WT *E. coli* cell after initial attachment. These images represent the position of the highlighted cell over time. There are many more frames between these time points, so the images do not represent the actual spin rate. The cell spun after it landed on top of a protruding line and before settled after 3 min (Bar = 2 μm). The cell body is highlighted with yellow line and the edges of the protruding line patterns are labeled with white dotted lines. (**b**) Fluorescence and SEM images of WT *E. coli* cells attached on top of 5 μm wide line patterns. The flagellum-like structures are indicated with arrow signs.

To further investigate the mechanism of preference in cell orientation on top of narrow line patterns, the attachment of WT *E. coli* cells were followed in real time after inoculation using fluorescence microscopy. While some cells settled right after landing on the top of 5 μm wide line patterns; the vast majority of cells contacted the surface with one pole first and then settled on top of line patterns after spinning several rounds. A series of time-lapse images of a representative cell is shown in Fig. 3a. The rotation patterns observed in our experiments are similar to that reported by Silverman *et al.*^42^, which showed that *E. coli* W3110 cell body can spin with a single flagellar filament (hook or polyhook) attached to a substratum. This finding along with the data of *fliC* and *motB* mutants suggests that flagella are involved in the adjustment of cell orientation described above.

*E. coli* has peritrichous flagella^38^. The rotation pattern of *E. coli* cells during attachment (Fig. 3a) suggests that the flagella close to one pole may play a key role in this process. To obtain more direct evidence, the WT *E. coli* cells were fluorescently labeled with Alexa Fluor® 594 NHS Ester (Succinimidyl Ester) to visualize flagella and monitor the attachment on top of 5 μm wide line patterns. The presence of flagella was also evidenced with SEM analysis of attached cells. The fluorescence and SEM images revealed that hair-like structures may be involved in the attachment of WT *E. coli* cells on top of the patterns (Fig. 3b). These structures appeared to be flagella because they were around 6 μm long (typical length of *E. coli* flagella^39^) and were only observed on WT *E. coli* cells and *fimA* mutant, but not on *fliC* mutant (Supplementary Fig. S7). The length of flagella of biofilm cells and if that changes with pattern dimension are unknown due to the challenge in measuring flagellar length in 3D.

To further corroborate this result and understand if polar flagella alone can cause such rotation, we characterized the cell orientation of a wild-type *Pseudomonas aeruginosa* strain, PA14, on top of 5 μm wide line patterns since *P. aeruginosa* only has a polar flagellum. As shown in Supplementary Fig. S8, similar results were obtained for PA14 cells with even stronger preference in perpendicular orientation (56.5 ± 2.2% cells) than *E. coli* (46.7 ± 6.8% cells). Consistent with the *E. coli* results, mutation of the *fliC* gene in *P. aeruginosa* PA14 also abolished the preference in perpendicular orientation on narrow line patterns (Supplementary Fig. S8), which further supports that flagella, especially polar flagella, may play important roles in deciding cell orientation on top of micron scale topographic features.

### Surface topography affects the rotation pattern of *E. coli* cells during attachment

**Figure 4:**
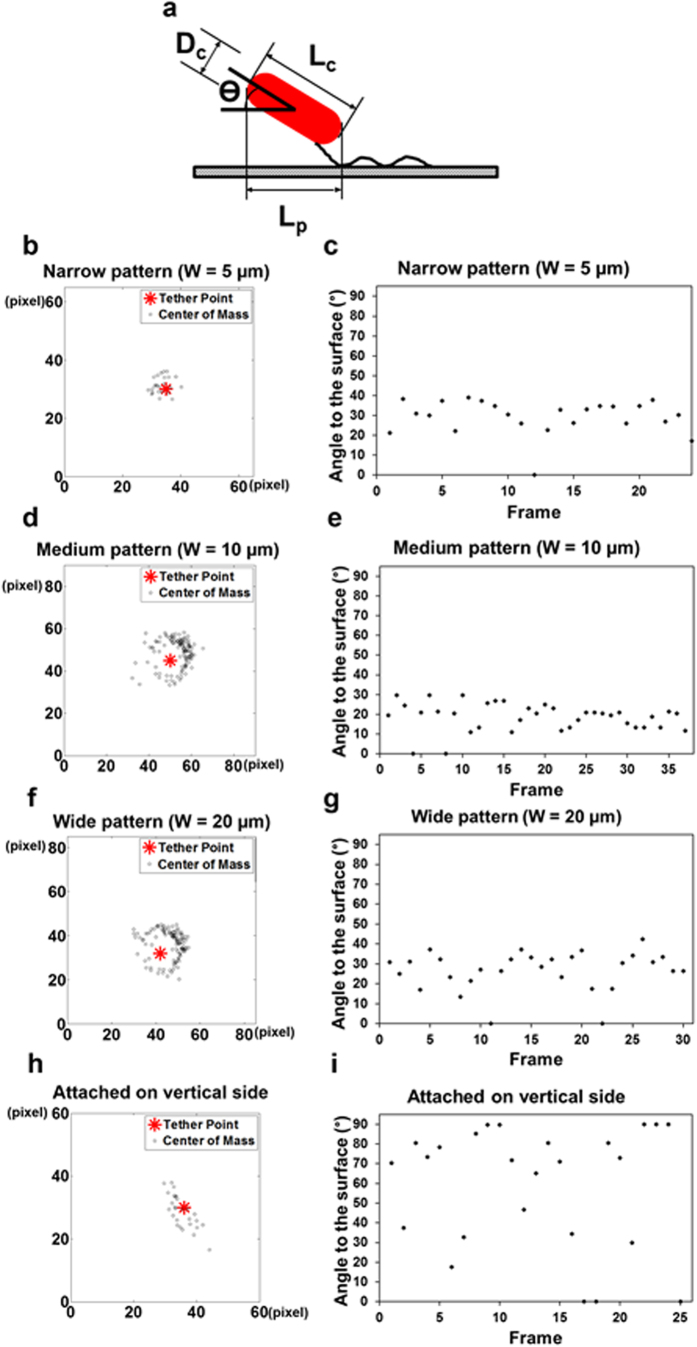
Rotation of WT *E. coli* cells on PDMS line patterns. (**a**) Definition of cell orientation during rotation. Rotational trajectory ((**b**,**d**,**f**,**h**) unit of x and y-axis is 0.16 μm/pixel) and the angle between cell body and the substratum surface (x-y plane) (**c**,**e**,**g**,**i**); e.g., (**b,c**) are shown for a cell tethered on the top of a narrow protruding line; (**d,e**) are shown for a cell tethered on the top of a line with medium width; (**f,g**) are shown for a cell tethered on the top of a wide line; (**h,i**) are shown for a cell tethered on the vertical side of a narrow line.

A striking discovery from our cell tracking is that the cells tethered on the vertical side of line patterns (regardless of pattern width) rotated differently compared to those on top of line patterns and flat PDMS surfaces (Fig. 4). It has been shown previously that *E. coli* cells attached to a flat surface rotate nearly in parallel to the tethered surface with a polar flagellum^43^. Such rotation is rather smooth with the cell track close to a round circle and the cell body aligned relatively parallel to the tethered surface (from zero to a small angle)^43^. In comparison, polar-flagellated *P. aeruginosa* PAO1 cells rotate with relatively large angles (0 to 90°) between cell body and the surface due to the flexibility of flagellar hook^43^. To better understand cell rotation patterns, the mass centers of cells in our system were tracked using Automated Contour-based Tracking for *In Vitro* Environments (ACT*IV*E)^44^. The coordinates of cell center on medium and wide line patterns during attachment fell into a circular shape (Fig. 4d,f and Supplementary Fig. S9), which is consistent with the cells rotating on flat surfaces as described in previous study using cell-tethering analysis^43^. To monitor the orientation of cell body during rotation, we characterized the angle between cell body and the substratum surface (top of topographic line patterns) using the projected cell length and the actual cell length (the longest in the series of cell images) (Fig. 3a). When the cells rotated on medium-width and wide line patterns, the orientation of cell body was relatively parallel to the substratum surface (Fig. 4e,g and Supplementary Fig. S10; Supplementary Movie S1). In comparison, the rotational trajectory of *E. coli* cells attached at the center of the narrow patterns appeared to be rather irregular compared to the cells on medium and wide patterns, although the orientation of cell body remained relatively parallel to the substratum surface (Fig. 4b,c). More striking differences were observed for the cells tethered on the vertical edge of patterns, which did not rotate in a circular trajectory but showed frequent and random change in orientation (Fig. 4h,i; Supplementary Movies S2). This is similar to *P. aeruginosa* PAO1 cells tethered to a flat surface^43^. In addition, the cells tethered on the vertical side of line patterns (regardless of pattern width) frequently reorientated the angle of cell body (position between 0 and 90°) during rotation (Fig. 4i and Supplementary Fig. S11). Collectively, *E. coli* cells attached on narrow patterns and on the vertical side of topographic patterns showed different rotation patterns compared to those tethered on smooth surfaces or on top of medium and wide patterns, and are more like the rotation pattern of *P. aeruginosa* with a single flagellum^43^. We speculate that these changes in the rotation pattern may cause the cell body to rotate off the regular rotation plane, contributing to the reduced adhesion and stress (below) of attached cells.

### Cell morphology on line patterns with different widths

**Figure 5:**
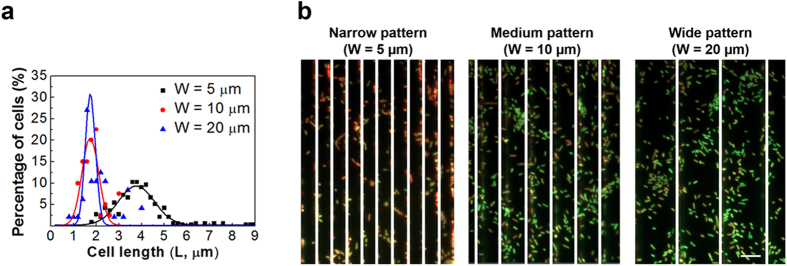
Cell morphology on narrow line patterns. (**a**) Length of attached WT *E. coli* cells on narrow (5 μm), medium (10 μm), and wide (20 μm) line patterns at 24 h after inoculation. (**b**) Representative fluorescence images of 24 h WT *E. coli* biofilm cells on 5 μm tall line patterns with 3 μm inter-pattern distance. Cells were labeled with acridine orange. The grooves are covered with white lines for the convenience of viewing cells on top of line patterns.

In addition to cell orientation, we also noticed that the length of attached cells varied with pattern width (Fig. 5a). The average cell length on narrow line patterns (W = 5 μm) was 4.0 ± 1.1 μm. This is about twice of the cell length on the medium (W = 10 μm) and wide (W = 20 μm) line patterns at 24 h (established biofilm) after inoculation, which was 1.9 ± 0.5 μm and 2.2 ± 0.7 μm, respectively. No significant difference in cell length was found between medium and wide line patterns (*p* > 0.05, one way ANOVA followed by Tukey test) and the cell length on these surfaces was comparable with that in 24 h biofilms on flat gold surfaces^25^. To better understand the difference in cell length, we also monitored the cells after 2 h of initial adhesion. The average cell length on top of 5 μm wide line patterns was found to be 5.3 ± 1.4 μm, similar to that on smooth PDMS surfaces (4.9 ± 1.49 μm) and planktonic cells collected from the same culture at the same time point (5.1 ± 2.5 μm; *p* > 0.05, one way ANOVA followed by Tukey test). Thus, the difference in cell length observed after 24 h of incubation was not due to selection of cells by the surfaces during attachment, but rather occurred during biofilm growth.

Because metabolic activities affect the size of bacterial cells^45^, we further compared the transcriptional activities of attached cells using acridine orange staining which shows green and red fluorescence when binding to DNA and RNA, respectively. As shown in Fig. 5b, cells on narrow line patterns after 24 h incubation had more RNA (stronger red fluorescence) compared to the cells on medium and wide line patterns, which indicates that the cells attached on narrow line patterns had higher transcriptional activities and thus possibly a higher level of overall gene expression. However, such activities did not lead to more growth (conversely, reduced biofilm mass and cell cluster formation was observed; see below) suggesting that narrow line topography may present an environmental stress to the bacterial cells, leading to decreased cell adhesion and biofilm growth.

### Topographic line patterns reduced cell cluster formation and biofilm formation

**Figure 6:**
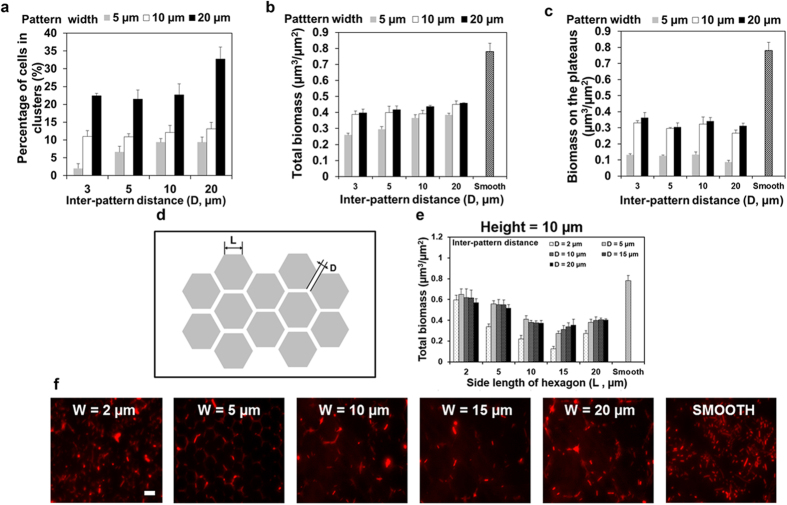
Cell cluster and biofilm formation of WT *E. coli* on PDMS surfaces with topographic patterns. (**a**) Cell clusters on patterns with varying width (5, 10, or 20 μm) and inter-pattern distance (3, 5, 10, or 20 μm). (**b**) Total biomass on flat PDMS and PDMS surfaces modified with line patterns. (**c**) Biomass on top of line patterns and flat PDMS (mean ± standard error shown; at least three biological replicates were tested for each sample). (**d**) Schematic of the hexagon shaped patterns. (**e**) Biomass of the WT *E. coli* biofilms on PDMS surfaces modified with 10 μm tall hexagon shaped patterns with varying side length (2, 5, 10, 15 or 20 μm) and inter-pattern distance (2, 5, 10, 15, or 20 μm) (mean ± standard error shown). (**f**) Representative images of the biofilms on PDMS surfaces modified with 10 μm tall hexagon shaped patterns with side length of 2, 5, 10, and 20 μm (with 2 μm inter-pattern distance), and smooth PDMS surface (Bar = 5 μm).

In addition to the effects on cell orientation, the topographic line patterns were also found inhibitory to cell attachment and cell cluster formation (Fig. 6). We defined a cluster as a group of six or more cells all within 1 μm of a neighboring cell. This relatively stringent criterion allowed us to focus on the cells that have close interactions. The percentage of cells in clusters was calculated by dividing the number of cells in defined clusters by the total cell number on the surface of interest. The results revealed that pattern width is positively correlated with cell cluster formation on top of line patterns (Fig. 6a; *r* > 0.75 for all patterns studied, Pearson correlation analysis, *p* < 0.0005). For example, the percentage of cells associated with cell clusters was 2.0 ± 3.2%, 11.0 ± 1.6%, and 22.3 ± 1.8% on 5, 10, and 20 μm wide patterns, respectively, when the inter-pattern distance was 3 μm (Fig. 6a). Cluster formation on the widest (20 μm) patterns tested with the largest inter-pattern distance (20 μm) was found to be 32.8 ± 8%, which is close to 30 ± 0.03% on flat PDMS. In comparison, inter-pattern distance (D = 3, 5, 10, or 20 μm; Fig. 1a) only showed influence on cell cluster formation on top of narrow patterns. The pattern with the narrowest width (5 μm) and shortest inter-pattern distance (3 μm) tested here reduced cluster formation to 2.0 ± 3.2% (14-fold reduction compared to the flat PDMS), indicating that narrow patterns with short inter-pattern distances can effectively reduce cell cluster formation.

Consistent with the reduction in cell cluster formation, the presence of line patterns reduced the total biomass (including biofilms on the top and side of patterns and in the grooves) on PDMS; e.g., the biomass was 0.78 ± 0.09 μm^3^/μm^2^ on smooth PDMS and 0.46 ± 0.005 μm^3^/μm^2^ on PDMS with 20 μm wide patterns and 20 μm inter-pattern distance, respectively (Fig. 6b). For any given inter-pattern distance, the total biomass of biofilms increased with pattern width. The inter-pattern distance (D = 3, 5, 10, or 20 μm; Fig. 1a) did not show major effects except for the pattern width of 5 μm. At this pattern width, the total biomass increased with inter-pattern distance (*r* > 0.85 for all patterns studied, Pearson correlation analysis, *p* < 0.0005). The biomass of biofilms on PDMS surfaces modified with 5 μm wide line patterns was 0.25 ± 0.02 μm^3^/μm^2^ and 0.38 ± 0.02 μm^3^/μm^2^ when the inter-pattern distance was 3 and 20 μm, respectively. Hence, even though the micron scale topography provided more surface area (due to the vertical edges), the 5 μm wide line patterns with 3 μm inter-pattern distance showed the most reduction (by 68%; 0.25 ± 0.02 μm^3^/μm^2^ vs. 0.78 ± 0.09 μm^3^/μm^2^) of biofilm formation compared with smooth PDMS surfaces (for the same projected area in x-y plane). The reduction of fouling was more evident on top of the line patterns. Specifically, 5 μm wide line patterns exhibited strong inhibition of biofilm formation, which was around 90% reduction compared to the same area of flat PDMS surfaces (Fig. 6c). The biomass of cells attached on top of line patterns increased with pattern width; e.g., the biomass was 0.12 ± 0.004 μm^3^/μm^2^ and 0.36 ± 0.06 μm^3^/μm^2^ on 5 and 20 μm wide patterns (both with 3 μm inter-pattern distance), respectively. The inter-pattern distance did not show significant effects. Collectively, the data indicate that narrow patterns are less prone to biofilm formation.

### New principles for rational design of antifouling surface topographies

Inspired by these results and our previous study^24^, we propose the following four principles for rational design of antifouling surface topographies. First, the height of topographic features should be at least 10 μm to prevent flagella from reaching into the grooves because the average length *E. coli* cell flagella is 6–10 μm^39^. Second, the area of plateaus should be smaller than 400 μm^2^ to prevent significant biofilm formation on top of the plateaus. The principle of this threshold surface area is based on our previous finding that *E. coli* adhesion on protruding square shaped patterns is significant only when the square is 20 μm × 20 μm or bigger^24^. Third, the gap between topographic features should be 2–5 μm to prevent bacteria from falling in the grooves or bridging over the gap. This is because most *E. coli* cells are 2–5 μm (previous report^25^ and our experience), which is also supported by the data that the strongest inhibition was observed with an inter-pattern distance of 3 μm in this study (Fig. 6b). Fourth, the shape of topographic features should have more edges or specific curvatures (e.g. hexagon shape vs. square shape) to minimize the interaction between the cells that get in grooves. This principle is proposed because cells attached on the vertical sides may experience more stress (based on acridine orange staining results shown above) and the geometry of topographic patterns can help prevent cell-cell contact and cell clusters formation in grooves.

To validate these four principles, we designed 10 μm tall hexagon shaped protruding features to further reduce biofilm formation using surface topography (Fig. 6d). The 24 h biofilm formation of WT *E. coli* cells on PDMS surfaces modified with protruding hexagon shaped patterns with heights (H) of 10 μm, side length (L) of 2, 5, 10, or 20 μm, and inter-pattern distance (D) of 2, 5, 10, 15, or 20 μm was tested (Fig. 6d). As shown in Fig. 6e,f, the biomass of 24 h biofilms formed on surfaces with hexagon shaped patterns with a side length of 15 μm and 2 μm spacing between patterns was 83.6 ± 2.9% less than flat PDMS (0.13 ± 0.02 μm^3^/μm^2^ vs. 0.78 ± 0.18 μm^3^/μm^2^; *p* < 0.0001, two way ANOVA followed by Tukey test).

To verify if the height of topographic features is important, the biofilm formation of the WT *E. coli* and its *fliC* mutant were tested on PDMS surfaces modified with 2, 5, or 10 μm tall hexagon shaped patterns (Supplementary Fig. S12). Consistent with the report from Friedlander *et al.*^19^, we found that 2 μm tall patterns promoted biofilm formation of the WT *E. coli* (Supplementary Fig. S12a). Similar but lesser effects were observed for 5 μm tall patterns (Supplementary Fig. S12b). In the absence of flagellar filament (*fliC* mutant), the promotion of biofilm formation on PDMS surfaces modified with 2 or 5 μm tall hexagon shaped patterns was not observed (Supplementary Fig. S12a,b). This evidence confirmed that flagella play important roles in bacterial response to surface topography and the topographic features need to be 10 μm or taller to overcome the effects of flagella.

## Discussion

As one of the promising strategies to prevent biofouling, the effects of surface topography on biofilm formation have been intensively studied recently^17^,^18^,^19^,^20^,^21^,^22^,^24^,^46^,^47^. However, how surface topography affects bacterial physiology and how bacteria respond to topographic features during biofilm formation are largely unknown. To better understand the underlying mechanism, we systematically compared adhesion and biofilm formation of *E. coli* on line patterns with varying width and inter-pattern distance in this study.

**Figure 7:**
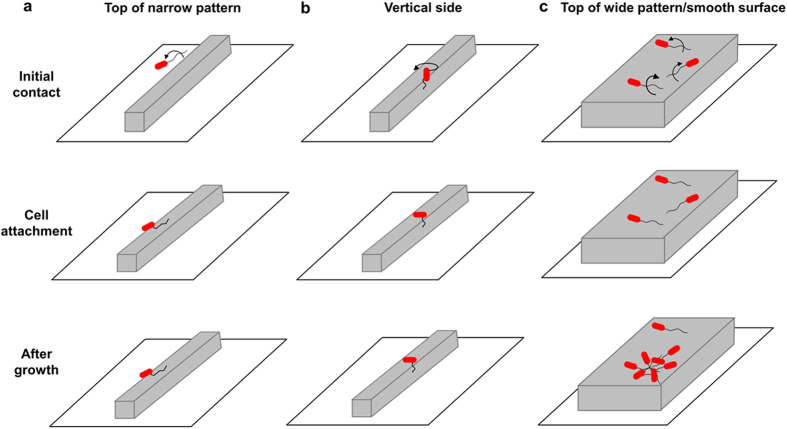
A hypothetical model of bacterial adhesion and cell cluster formation on top of protruding line patterns. Cells contact the top of narrow patterns (**a**), vertical sides (**b**), and top of wide patterns/smooth surfaces (**c**) exhibit different alignment in cell orientation, which further affects cell cluster formation. Only one flagellum is shown for each cell for clarity. The cells attached on the side of producing patterns and in the grooves are not shown.

*E. coli* cells were found to have a preference in alignment on top of protruding narrow line patterns. Based on real-time imaging and comparison between the WT *E. coli* and its isogenic mutants, flagella were found important to this newly observed phenomenon. We propose the following hypothetical model to explain the observed results. As shown in Fig. 7, when an *E. coli* cell approaches a solid surface, the cell makes initial surface contact with outer membrane appendages such as flagella or pili. On top of medium and wide line patterns or smooth surfaces, flagella can readily tether to the top of line patterns (Fig. 7c). Cells rotate regularly in a circular pattern and thus land with a random angle, leading to a uniform distribution of cell orientation. On narrow patterns; however, the available area on top of line patterns is limited. For a cell to attach on top of a narrow protruding line pattern, the flagella need to rotate rather perpendicularly to the orientation of the line and hit the top of the line. This will cause the cell to orientate in parallel to the line pattern. Because the lines are narrow, such events are infrequent leading to a low percentage of cells aligned in parallel to the line patterns (Fig. 7a). In comparison, the chance of bacterial flagella hitting the vertical side of narrow patterns is much higher because each line has two vertical sides and thus twice of contact area (D = W = 5 μm; Fig. 7b). After the initial attachment, the cell can use flagella to spin as shown in Fig. 7. The difference in tether point may alter the mechanical force that the cell experiences and consequently the rotation pattern and cell orientation after attachment. This is supported by the defects observed for the *motB* mutant. Following the settling of bacterial cell body, fimbriae may be involved in further adjustment of cell orientation. Further studies to visualize fimbriae directly will help reveal their role in this process. Because the average length of WT *E. coli* cells on top of narrow PDMS line patterns in this study is 4.0 ± 1.1 μm and the width of narrow PDMS surfaces is 5 μm, newly divided cells may have to actively readjust their position to ensure the maximum contact with the plateaus (Fig. 7b). This can possibly contribute to the higher level of stress among cells attached on narrow patterns (based on acridine orange staining) and reduced cell cluster formation.

Flagella are well known surface appendages that allow bacterial cells to move and make initial contact with a solid surface during biofilm formation by overcoming the long range repulsive force along the surface^39^,^40^. Recently, bacteria have also been found to use flagella to adapt to or overcome nano or submicron scale surface topography^9^,^19^,^23^. Using short (120 nm tall, 550 nm wide, and 750 nm spacing) topographic lines, Diaz *et al.*^23^ showed that *P. fluorescens* uses flagella to make contact with neighboring cells to facilitate cell-cell interaction. Using shallow pores or wells (20 or 200 nm in depth), Hsu *et al.*^9^ found that *E. coli*, *P. fluorescens*, and *Listeria innocua* use flagella or other cell outer membrane appendages to build a web on modified silica or alumina surfaces and form biofilms. Friedlander *et al.*^19^ reported that flagella are used by *E. coli* cells to adhere to PDMS surfaces modified with an array of hexagonal features (2.7 μm in height and 3 μm in diameter) and overcome these unfavorable surface topographies by exploring the extra surface. The flagellar length of planktonic *E. coli* cells ranges from 6 to 10 μm^39^. Given the important roles that flagella play during surface attachment, creating topographic features that are 10 μm or taller can help reduce fouling.

Interestingly, the average cell length on top of narrow line patterns was twice of that on medium and wide patterns. The longer cells also exhibited stronger transcriptional activities based on acridine orange staining. The width of narrow pattern was 5 μm, which is similar to the average cell length in 24 h biofilms on narrow line patterns. With more cells aligned perpendicularly to the orientation of line patterns, the narrow line patterns reduced cell cluster formation, which also contributed to the reduction of total biofilm formation. We observed that cell cluster development under our experimental condition involved both the proliferation of attached cells and recruitment of other cells to the location (data not shown). Further study is needed to understand how surface topography affects the growth of biofilm cells, which is part of our ongoing work.

In summary, we investigated the effects of surface topography on cell orientation, cluster formation, and biofilm formation of *E. coli* on top of 5 μm tall line patterns with varying width and inter-pattern distance. The data revealed that *E. coli* cells prefer to align perpendicularly to the direction of narrow line patterns. As the line patterns got wider, the orientation of cells became more random; and cluster formation and cell density increased towards those on smooth PDMS surfaces. The genes *fliC*, *motB*, and *fimA* were found important to the observed preference in cell orientation. In addition, the cells on narrow patterns were found longer and had higher transcriptional activities. These findings complement previous studies^21^,^24^,^46^,^48^,^49^ and provide new evidence that bacteria do “read the map” during biofilm formation. These data shed new light on the mechanistic understanding of biofilm formation and can help rational design of better nonfouling surfaces, as we demonstrated with 10 μm tall hexagon shaped patterns. This study is based on pure cultures of *E. coli*. Further test of new topographies for biofilm formation of both motile and non-motile bacterial pathogens will help develop better biomaterials and medical devices. Additional improvement may be possible through synergy with other mechanisms, e.g. the Cassie-Baxter state or synergy with antimicrobials^50^,^51^.

## Methods

### Bacterial strains and growth medium

*E. coli* RP437/pRSH103^31^ is a motile and chemotaxis wild-type (WT) strain and referred as WT *E. coli* in this study. This strain and its four isogenic mutants (*fliC*, *motB*, *fimA*, and *luxS*; Supplementary Table S1) were used to investigate the attachment on top of PDMS line patterns. *E. coli* RP437 was chosen because it is a model strain for biofilm research and allows us to compare with our previous results of this strain^24^,^31^,^52^. The plasmid pRSH103 carries the *dsRed* gene, which labels the cells with constitutively expressed red fluorescent protein. All *E. coli* cells were routinely grown at 37 °C with shaking at 200 rpm in Lysogeny Broth (LB)^53^ consisting of 10 g/L NaCl, 5 g/L yeast extract, and 10 g/L tryptone. Tetracycline was supplemented at a concentration of 30 μg/mL (henceforth LB-Tet) when needed to help keep plasmid.

A *P. aeruginosa* wild-type strain, PA14, and its two isogenic mutants (*fliC* and *pilB*) taken from the PA14 transposon insert mutant library^54^ were also used to study the orientation of attached cells on top of 5 μm wide PDMS line patterns. *P. aeruginosa* strains were grown at 37 °C with shaking at 200 rpm in LB.

### Strain construction

*E. coli* RP437 Δ*fliC* (*E. coli* RHG01) and RP437 Δ*fimA* (*E. coli* RHG02) were constructed using the λ red recombination system^55^. Briefly, the λ red recombination system on plasmid pKM208 was used to replace the target gene on the chromosome of *E. coli* RP437 with the polymer chain reaction (PCR) products containing kanamycin resistance marker flanked with ~700 bp of genomic sequence from each side of the target gene. The primers used are listed in Supplementary Table 2 and the genomic DNA of JW4277 (Δ*fimA::kan*) and JW1908 (Δ*fliC::kan*) from the Keio collection^56^ was used as templates. Before the PCR products were electroporated into *E. coli* RP437 cells, the plasmid pKM208 was transformed into the *E. coli* RP437 cells. *E. coli* RP437/pKM208 cells were grown at 30 °C with 1 mM IPTG and 100 μg/mL ampicillin to promote the production of Red and Gam proteins^55^. Gene deletion was verified by PCR using one primer upstream of the target gene and another primer within the drug marker (Supplementary Table S2; result not shown). The plasmid pKM208 was cured after gene deletion by growing the mutants at 42 °C.

### Genetic complementation of the *motB*, *fliC*, and *fimA* mutants

The isogenic mutants of *motB*, *fliC*, and *fimA* genes of the WT *E. coli* were complemented by cloning corresponding genes and their native ribosome binding site into the plasmid pRSH103^52^. DNA fragments that contain the genes and their native ribosome binding site were amplified using primers listed in the Supplementary Table S3 with the chromosome DNA of WT *E. coli* as template. The PCR products were inserted into the vector pRSH103 between *Hind*III and *Spe*I for *fimA* and *motB* genes or using a single restriction site (*Hind*III) for the gene *fliC*. Isopropyl β-D-1-thiogalactopyranoside (IPTG) was added at 1 mM into the culture to induce gene expression (controlled by a *lac* promoter).

### Preparation of surfaces with topographic patterns

Microfabrication of PDMS surfaces was achieved through photolithography and soft lithography by following previously described procedures^57^,^58^,^59^ with slight modifications. For all line shaped PDMS patterns, pattern length (L) was fixed at 4 mm and pattern height (H) was fixed at 5 μm. The pattern height was set to be 5 μm because it is relatively easy to prepare and still high enough to separate cells on top of patterns from those in grooves. The line patterns were designed to have width (W) of 5, 10, or 20 μm, and inter-pattern distance (D) of 3, 5, 10, or 20 μm (Fig. 1a). The hexagon shaped patterns were designed to have height (H) of 2, 5, or 10 μm, side lengths (L) of 2, 5, 10, 15, or 20 μm, and inter-pattern distance (D) of 3, 5, 10, or 20 μm (Fig. 6d). The topographic features were created by using photolithography to etch silicon wafers with complementary patterns at the Cornell NanoScale Science & Technology Facility (Cornell University, Ithaca, NY, USA). Soft lithography was used to prepare the topographic PDMS patterns. Briefly, PDMS elastomer base and curing agent from Sylgard^®^184 Silicone Elastomer Kit (Thermo Fisher Scientific, Waltham, MI, USA) were mixed at a 10:1 ratio (approximately 15 g of PDMS per set of patterns) and degassed for 30 minutes. The solution was poured slowly onto the silicon wafer and left to cure for 24 h at 50 °C, followed by another 24 h to settle at room temperature. The PDMS patterns were then carefully peeled off the silicon wafers and individually cut and stored in petri dishes for later use in experiments.

### Biofilm formation

To study the orientation of attached cells and formation of cell clusters in biofilms formed on top of PDMS line patterns, overnight cultures of the WT *E. coli* and its four isogenic mutants were used to inoculate biofilm medium (20 mL of LB medium supplemented with 30 μg/mL tetracycline) in petri dishes containing sterilized PDMS surfaces to an initial optical density at 600 nm (OD_600_) of 0.05. Biofilm cultures of PA14 and its two isogenic mutants were prepared in the same way in LB medium without antibiotics. Patterned PDMS surfaces were sterilized by soaking in 190 proof ethanol for 30 min and then dried in a clean petri dish for 40 min at 50 °C before inoculation. To study bacterial adhesion and biofilm formation on face-down pattern surfaces, the sterilized PDMS patterns were put upside down (with each end supported by a piece of microscope glass slide) in a petri dish containing 20 mL LB medium supplemented with 30 μg/mL tetracycline. Biofilm cultures were incubated at 37 °C for 2 or 24 h without shaking. Bacterial attachment on top of 5 μm wide line patterns in static solutions was followed using an Axio Imager M1 fluorescence microscope (Carl Zeiss Inc., Berlin, Germany) with a 63× dry objective lens for acquiring RFP and bright field images. Images and real time movies (for the WT *E. coli* on 5 μm wide line patterns) were recorded and processed using the ZEN 2.0 software (Carl Zeiss Inc., Berlin, Germany).

To study the settlement of the WT *E. coli* cells on top of line patterns, cells from an overnight culture were pretreated with 10 μg/mL chloramphenicol (Sigma Aldrich, St. Louis, MO, USA) for 1 h at 37 °C with shaking at 200 rpm and then used to inoculate biofilm cultures as described above.

### Microscopy

The PDMS surfaces with biofilms were gently washed three times with 0.85% wt/vol NaCl solution to remove planktonic cells. WT *E. coli* biofilms were imaged immediately after washing. To study the orientation of bacterial cells on top of topographic line patterns, 2D fluorescence images were taken by focusing on the top of protruding lines. For calculating biomass, 3D information was obtained from a series of z stack images (1 μm interval), which were analyzed using the software COMSTAT^60^. *P. aeruginosa* biofilms were further labeled with acridine orange (Sigma Aldrich, St. Louis, MO, USA) before imaging. To do this, the PDMS surfaces with biofilm cells were soaked in 3 mL acridine orange solution (0.5 mg/mL) for 5 min and gently washed three times in 0.85% NaCl solution to remove excessive dye. To study the cellular activity of WT *E. coli* biofilm cells on PDMS surfaces modified with line patterns, 24 h WT *E. coli* biofilms were also labeled with acridine orange as described above. All biofilms were visualized using an Axio Imager M1 fluorescence microscope with a 63× dry objective. At least five positions on each pattern were randomly selected for imaging. Each condition was tested with three replicates.

### Image analysis

Microscopic images were processed using the ZEN 2.0 software and further analyzed using Adobe Photoshop CS5.1 to determine the orientation and clustering of bacterial cells attached on the line patterns. The orientation of each attached cell was measured by comparing to the orientation of the line pattern it is attached to. The axis perpendicular to the orientation of line patterns was defined as 0°. Thus, the data of cell orientation were categorized as Perpendicular (0–30°), Diagonal (30–60°), or Parallel (60–90°) (Fig. 1b). Surface coverage and biomass of biofilms were quantified using the software COMSTAT^60^.

### Flagella imaging

The WT *E. coli* RP437 cells and its isogenic mutants *fliC* and *fimA* were fluorescently stained with Alexa Fluor® 594 NHS Ester (Succinimidyl Ester; Life technologies, Carlsbad, CA, USA) as described previously^61^. Briefly, an overnight culture of each strain was diluted 100 times into 20 mL LB medium and grown to mid-exponential phase. The bacterial cells from 1 mL culture were washed three times at room temperature by centrifugation (1000 × g, 15 min) and gently resuspended in 0.5 mL of motility buffer (0.01 KPO_4_, 0.067 M NaCl, 10^−4^ M EDTA [pH 7.0]). Alexa Fluor® 594 NHS Ester (Succinimidyl Ester, 10 mg/mL in dimethyl sulfoxide (DMSO)) was added into the final suspension of bacteria to a concentration of 200 μg/mL. Sodium bicarbonate (25 μL [1M]) was added to adjust the pH to 7.8. The labeling was conducted at room temperature in the dark for 1.5 h. Excessive dye was removed by washing with motility buffer. The fluorescently labeled WT *E. coli* cells were used to inoculate 5 mL LB in a clean petri dish to OD_600_ of 0.05 with sterilized PDMS surfaces modified with 5 μm wide line patterns at the bottom to follow bacterial adhesion. The PDMS surfaces were then washed three times with 0.85% NaCl after 2 h incubation at 37 °C in the dark. Cells were imaged with fluorescence microscopy (Imager M1 and Observer Z1 microscopes; Carl Zeiss Inc., Berlin, Germany) using a Hamamatsu ORCA-FLASH4.0LT camera (Hamamatsu Photonics, Hamamatsu, SZK, Japan) and a 63x oil lens (Carl Zeiss Inc., Berlin, Germany).

### Imaging with a scanning electronic microscope (SEM)

After 2 h biofilm formation at 37 °C, WT *E. coli* biofilms on PDMS surfaces modified with 5 μm wide line patterns were fixed with 2.5% glutaraldehyde in phosphate-buffered saline (PBS) for 30 min. Then, the PDMS surfaces with attached cells were coated with osmium tetroxide for 30 min and dehydrated with a series of 50, 75, 90, 95, and 100% proof ethanol (15 min each). Finally, the surfaces were treated with tetramethylsilane (TMS) and then sputter coated with an Au/Pd target for 60 s at 20 mA (Desk V HP, Denton Vacuum LLC, USA). High resolution SEM images were acquired using LEO 1550 FESEM at Cornell Center for Materials Research (CCMR, Ithaca, NY, USA).

### Analysis of cell rotation using modified ACT*IV*E

Time-lapse videos were cropped to highlight a cell of interest for analysis. To investigate the effects of surface topography on rotation patterns, Automated Contour-based Tracking for *In Vitro* Environments (ACT*IV*E) was employed^44^, with the following modifications. First, time-lapse videos were segmented. Briefly, each frame was individually processed using a contour based intensity profile map, and individual cells were identified based on contour information and fit with an ellipse for tracking the center of mass. To link information of interest frame to frame, only the center of mass for the cell closest to the image midpoint was recorded. All other cell information was discarded to limit issues associated with cells flowing through the time-lapse field of view. Center of mass behavior was manually checked by overlaying the ellipse fit information on the raw time-lapse images. Once an appropriate fit was verified for all frames, a single tether point was selected based on an overlay of the phase and time-lapse fluorescence images.

### Orientation of cell body

The angle between the cell body and tethered surface was calculated based on cell images during attachment. The cell body was assumed to be cylindrical shape. θ is the angle between the cell body and tethered surface (Fig. 4a).


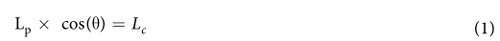


where L_c_ is the length of cell body when cell settled and L_p_ is the projected length of cell body during cell rotation. The length of *E. coli* cells, the projected length of cell body, and the diameter of cell body on each frame of the real time images were measured using the AxioVision Miscosopy Software (Carl Zeiss Inc., Berlin, Germany).

### Statistical analysis

All statistical analyses were performed by using SAS 9.1.3, Windows version (SAS, Cary, NC, USA). Results with *p* < 0.05 were considered statistically significant.

## Additional Information

**How to cite this article**: Gu, H. *et al.* How *Escherichia coli* lands and forms cell clusters on a surface: a new role of surface topography. *Sci. Rep.* **6**, 29516; doi: 10.1038/srep29516 (2016).

## Supplementary Material

Supplementary Movie S1

Supplementary Movie S2

Supplementary Information
