# Supplementary material for: How Escherichia coli lands and forms cell clusters on a surface: a new role of surface topography
Source: Sci Rep. 2016 Jul 14;6:29516. doi: 10.1038/srep29516 (PMC4944170; doi:10.1038/srep29516)
Supplement: Supplementary Information [file srep29516-s3.pdf]

Supplementary Information

for

How *Escherichia coli* lands and forms cell clusters on a surface: a new role of surface topography

Huan Gu[1, 2, *], Aaron Chen [1, 2, *, †], Xinran Song[1, 2], Megan E. Brasch[1,2], James H. Henderson[1,2], and Dacheng Ren[1,2,3,4]

[1]Department of Biomedical and Chemical Engineering, Syracuse University, Syracuse, NY 13244, USA.
[2]Syracuse Biomaterials Institute, Syracuse University, Syracuse, NY 13244, USA.
[3]Department of Civil and Environmental Engineering, Syracuse University, Syracuse, NY 13244, USA.
[4]Department of Biology, Syracuse University, Syracuse, NY 13244, USA.

Corresponding author:
Dacheng Ren: Phone 001-315- 443-4409. Fax 001-315- 443-9175. Email: dren@syr.edu

[*]These authors contributed equally

[†] Current address: Department of Mathematics, 310 Malott Hall, Cornell University, Ithaca, NY 14853, USA.

Table S1. List of bacterial strains and plasmids used in this study.

Strain	Genotype	Characteristics	Source/References
***E. coli* strains**			
RP437	Wild type [*thr*-1 *leuB6*(Am) *hisG*4(Oc) *fhuA*31 *metF*159(Am) *thiE1 rfbC*1 *eda*-50 *rpsL*136(strR) *araC*14 *mtl*-1 *xylA*5 λ^- *tsx*-78 *lacY*1 F-]	Wild type strain for biofilm study	1
RP3087	RP437 *(motB)580*	Motility mutant (point mutation in the *motB* gene)	2
KX1485	RP437 Δ*luxS::*Cm^r	Quorum sensing mutant, unable to synthesize AI-2	3,4
RHG01	RP437 Δ*fliC::*Kan^r	Unable to synthesize the subunits of flagella	This study
RHG02	RP437 Δ*fimA::*Kan^r	Unable to synthesize the subunits of fimbriae	This study
***P. aeruginosa* strains**			
PA14	Wild type [Human clinical isolate; Rif^r]	Wild type strain for biofilm study	5,6
PA1092	PA14 (*fliC*)	Flagellin type B transposon insertion mutant	7
PA4526	PA14 (*pilB*)	Type 4 fimbrial biogenesis protein PilB transposon insertion mutant	7
**Plasmids**			
pRSH103	*rfp,* Tet^r	To label the cells with constitutive red fluorescence for imaging	8
pRHG03	*motB*$^+$, Tet^r	pRSH103 with *motB* gene controlled by *lac* promoter	This study
pRHG04	*fliC*$^+$, Tet^r	pRSH103 with *fliC* gene controlled by *lac* promoter	This study
pRHG05	*fimA*$^+$, Tet^r	pRSH103 with *fimA* gene controlled by *lac* promoter	This study

Table S2. Primers used for constructing the *E. coli* mutants.

Target gene	Forward primer (5'-3')	Reverse primer (5'-3')
fimA	ACCGTTCACCCGTTACGTTT	TGCTCTTGTTTTTGCCCTGC
fliC	CGCCACTCATCGTAGGAGAA	CCGAGACCTCCATACCGTTG

Table S3. Primers used for complementing the *E. coli* mutants.

Target gene	Forward primer (5'-3')	Reverse primer (5'-3')
motB	ATTTGCGGCCGCTGTGCGTGCGGTGAAAAATC	GGACTAGTCATGTCAGCCAACAGTTCGTC
fliC	ATTTGCGGCCGCTAGCGGGAATAAGGGGCAGAG	ATTTGCGGCCGCAGTCTCAGTTAATCAGGTTACAACG
fimA	ATTTGCGGCCGCGCCAGTAATGCTGCTCGTTTT	GGACTAGTTTATCGCACAAGGGTGGGC

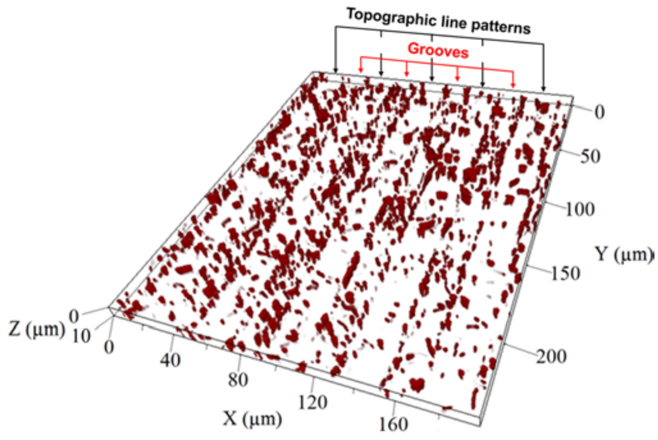

Figure S1. Representative 3D fluorescence image of WT *E. coli* biofilm. The image shows a PDMS surface modified with 20 µm wide topographic line patterns with 20 µm inter-pattern distance. The cells on top of protruding line patterns (5 µm tall) are the focus of this study.

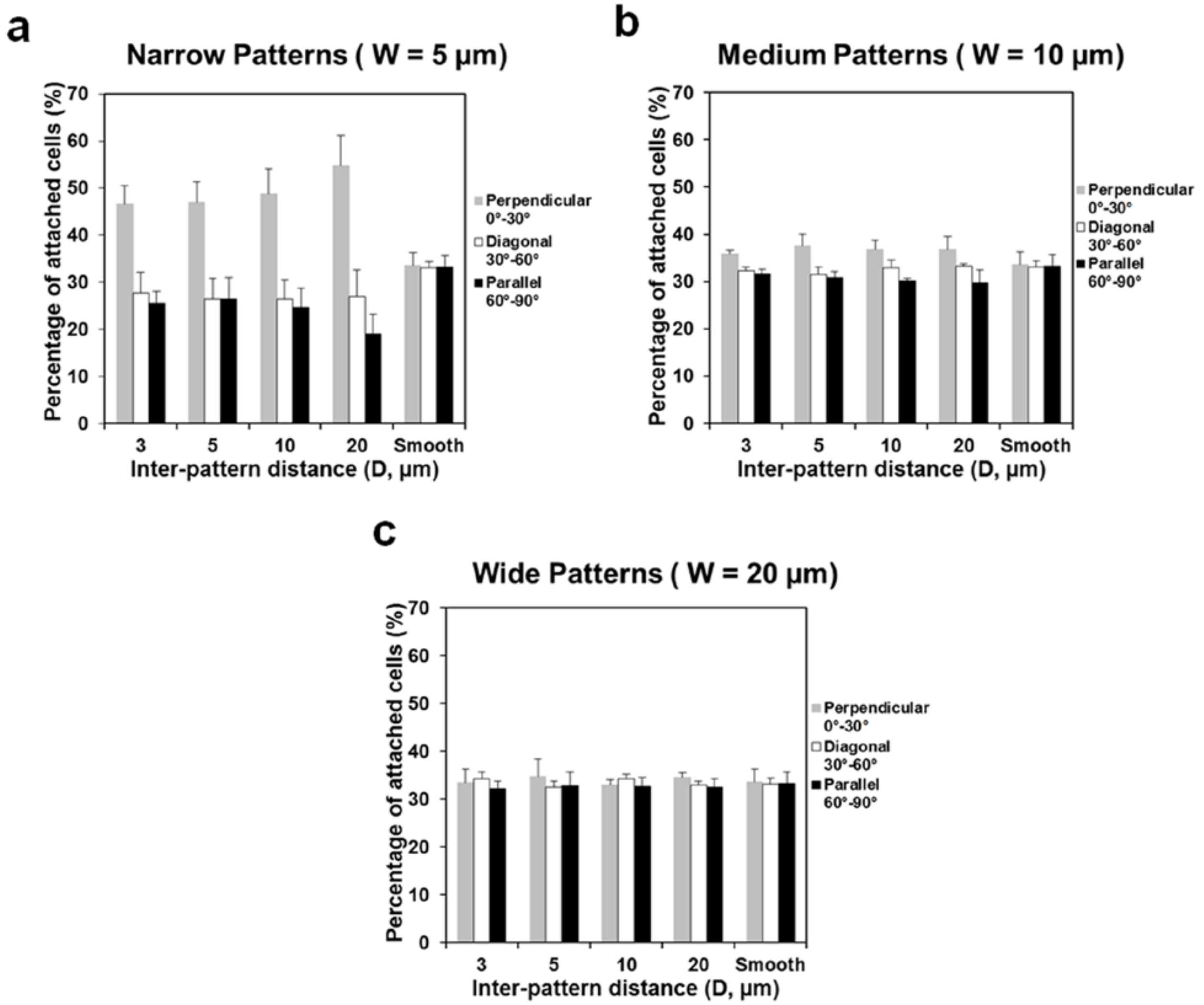

Figure S2. Orientation of the WT *E. coli* cells attached on top of PDMS line patterns at 24 h after inoculation (mean ± standard error shown; at least three biological replicates were tested for each sample). Distribution of cell orientation on top of (**a**) narrow (5 µm wide), (**b**) medium (10 µm wide), and (**c**) wide (20 µm wide) PDMS line patterns.

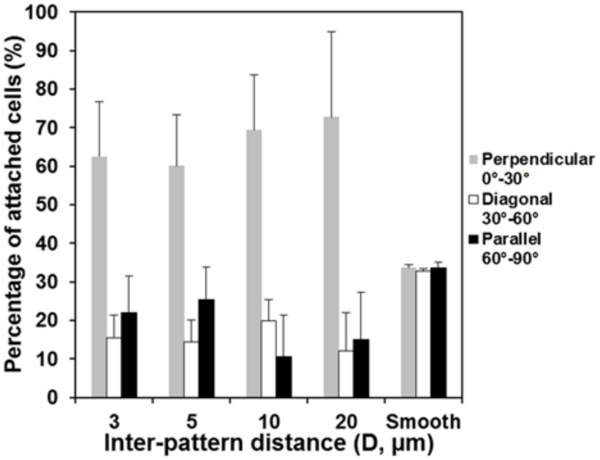

Figure S3. Orientation of WT *E. coli* cells on top of 5 μm wide line patterns and smooth PDMS surfaces after 2 h of attachment (mean ± standard error shown; at least three biological replicates were tested for each sample).

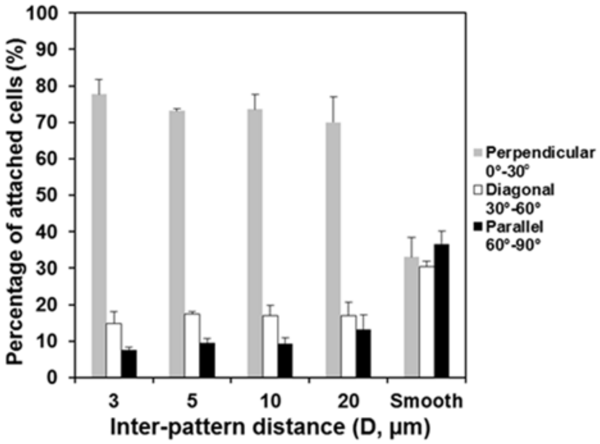

Figure S4. Orientation of WT *E. coli* cells on top of face-down 5 μm wide line patterns and smooth PDMS surfaces at 24 h after inoculation (mean ± standard error shown; at least three biological replicates were tested for each sample).

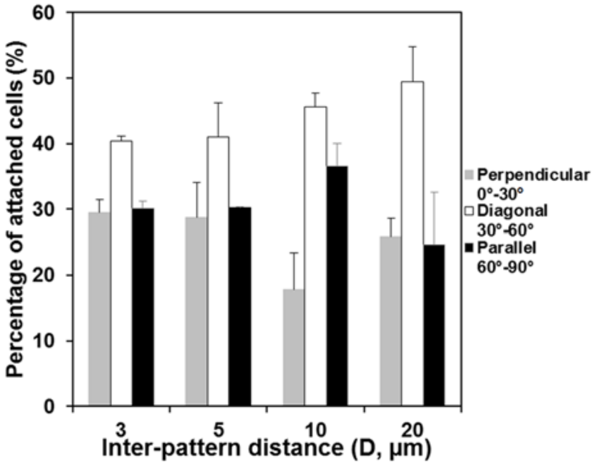

Figure S5. Orientation of WT *E. coli* cells on top of 5 µm wide line patterns after 24 h of culturing (mean ± standard error shown; at least three biological replicates were tested for each sample). Seeding cells were pretreated with 10 µg/mL chloramphenicol for 1 h before inoculation.

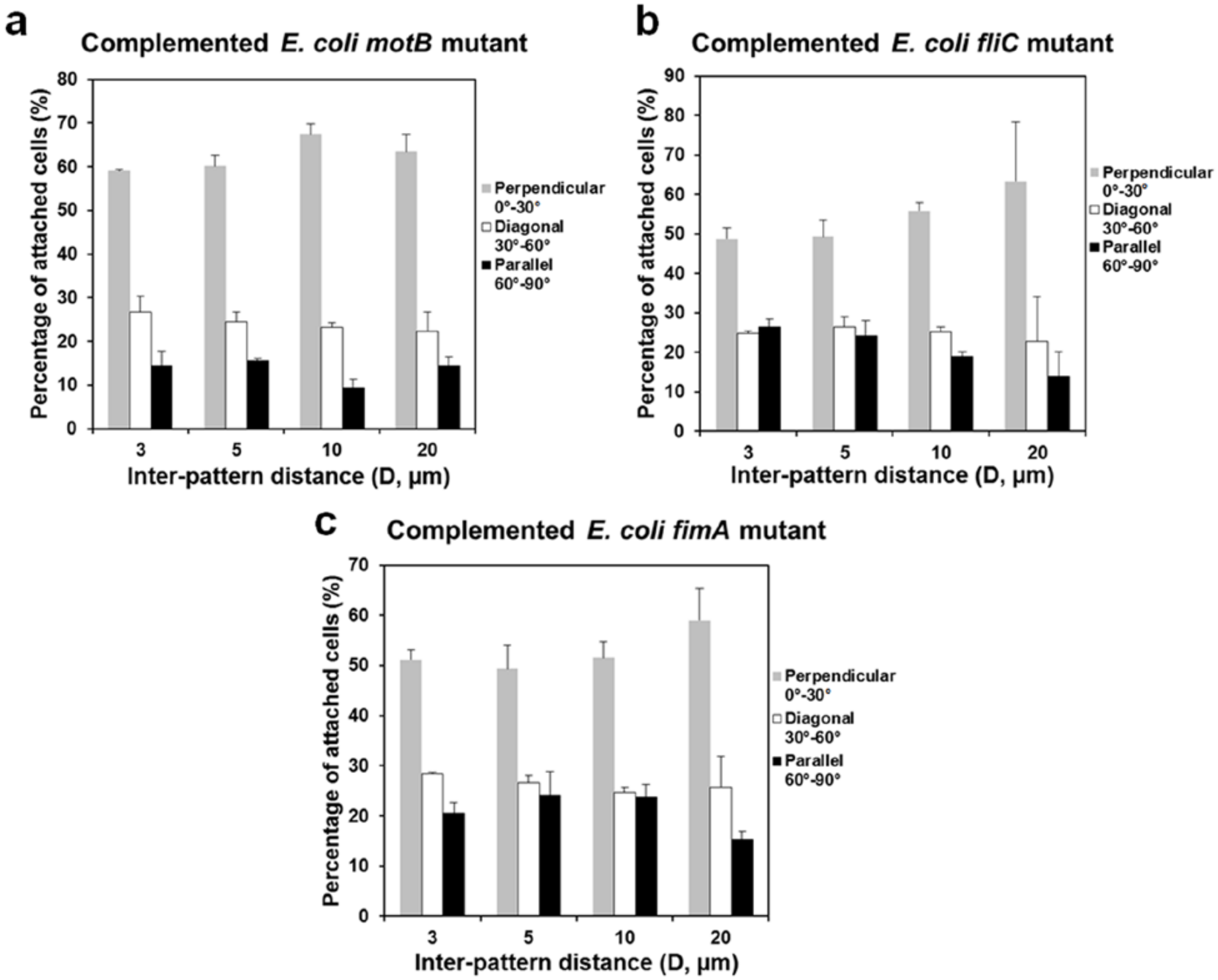

Figure S6. Distribution of cell orientation on top of 5 μm wide line patterns (mean ± standard error shown; at least three biological replicates were tested for each sample). (**a**) The complemented *motB* mutant *E. coli* RP3087/pRHG03. (**b**) The complemented *fliC* mutant *E. coli* RHG01/pRHG04. (**c**) The complemented *fimA* mutant *E. coli* RHG02/pRHG05.

.

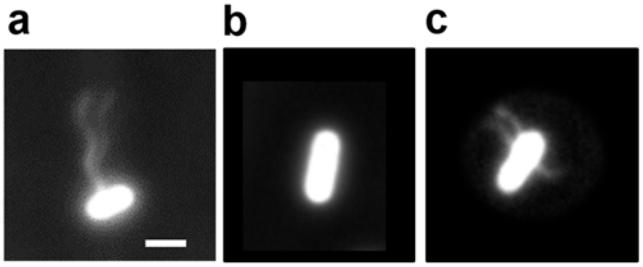

Figure S7. Fluorescence images of the planktonic cells showing flagella-like structures. The images show the cells of the WT *E. coli* (**a**) and its mutants of *fliC* (**b**) and *fimA* (**c**) genes. The cells harvested from planktonic cultures (OD_{600} = 0.6) were stained with Alexa Fluor® 594 NHS Ester (Succinimidyl Ester) Bar = 2 µm.

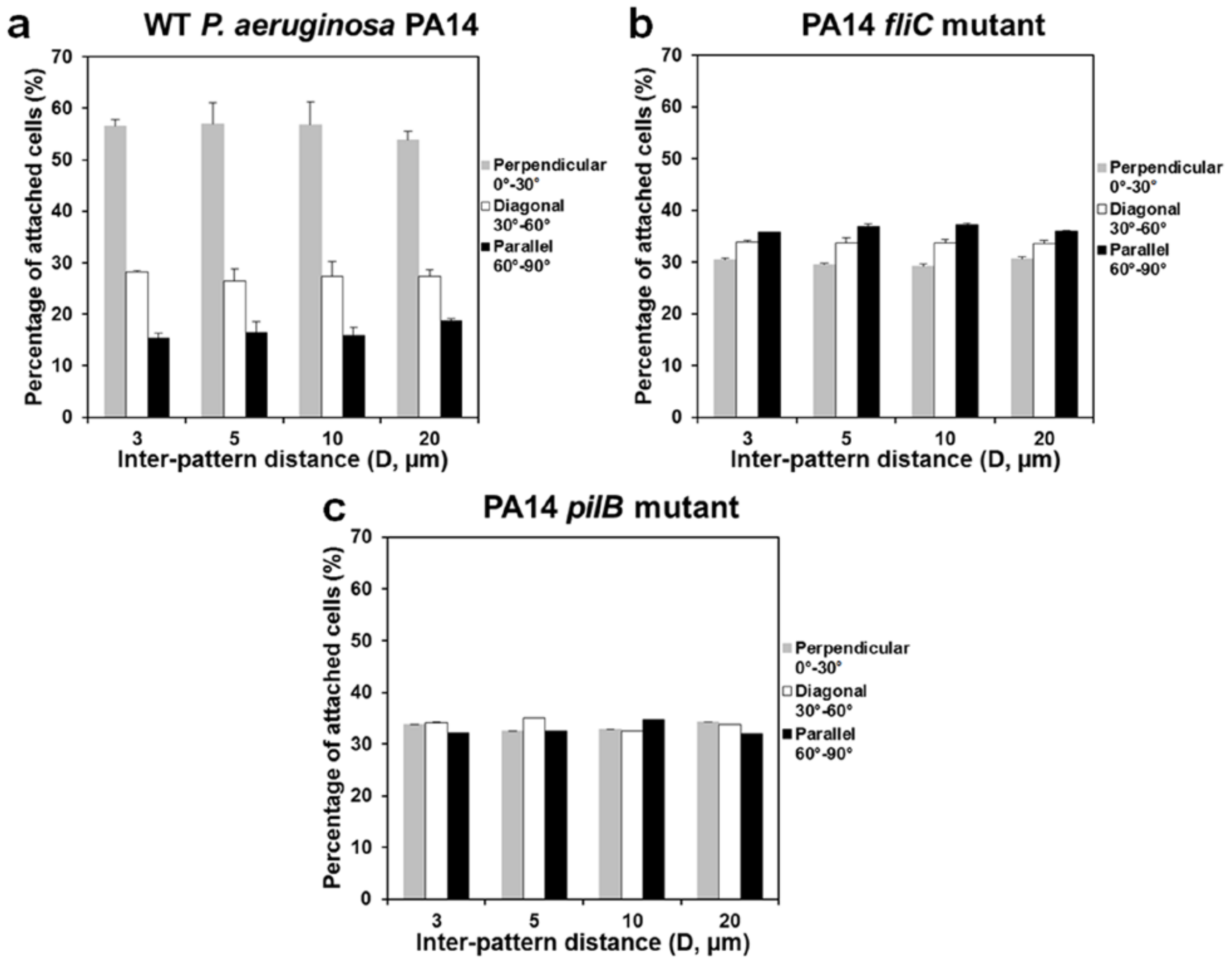

Figure S8. Distribution of cell orientations on top of 5 µm wide line patterns (mean ± standard error shown; at least three biological replicates were tested for each sample). (**a**) Wild-type *P. aeruginosa* strain PA14. (**b**) Δ*fliC* mutant of *P. aeruginosa* PA14 (PA1092). (**c**) Δ*pilB* mutant of *P. aeruginosa* PA14 (PA4526).

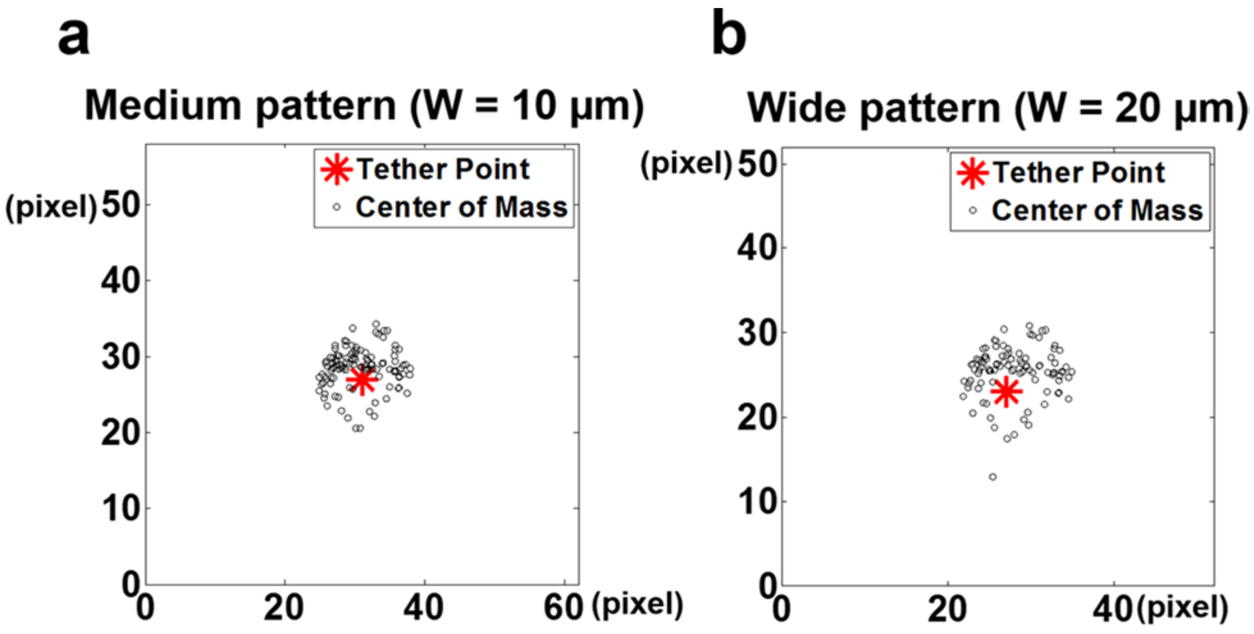

Figure S9. Rotation trajectories of cells tethered on top of medium (a) and wide (b) line patterns. The unit of x and y-axis is 0.16 μm/pixel. The images were taken with 1.6 s interval between every two frames.

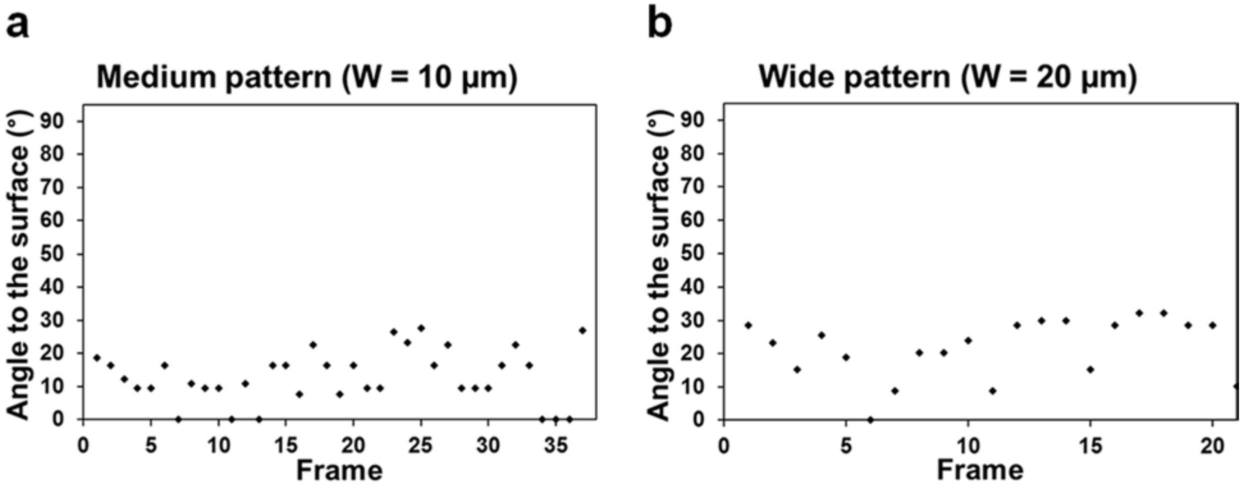

Figure S10. **The angle between cell body and the substratum surface (x-y plane) of WT *E. coli* cells tethered on the top of medium (a) and wide (b) line patterns.** The images were taken with 1.6 s interval between every two frames.

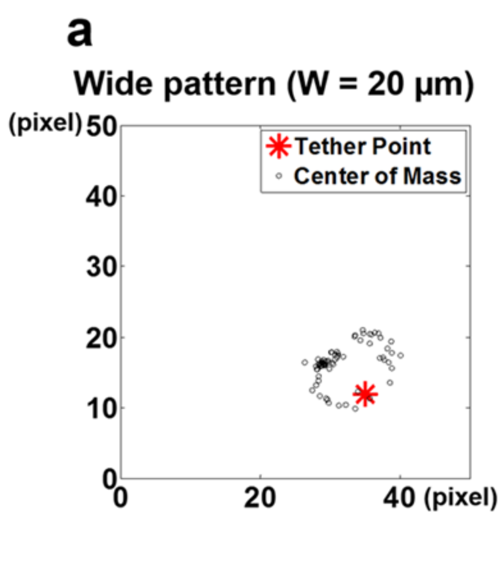

Figure S11. **Rotation trajectory (a; unit of x and y-axis is 0.16 μm/pixel) and the angle between cell body and the substratum surface (x-y plane) (b) of a cell tethered on the vertical side of a wide line pattern.** The cell rotation pattern was still a circle but was not centered around the tether point. The images were taken with 1.6 s interval between every two frames.

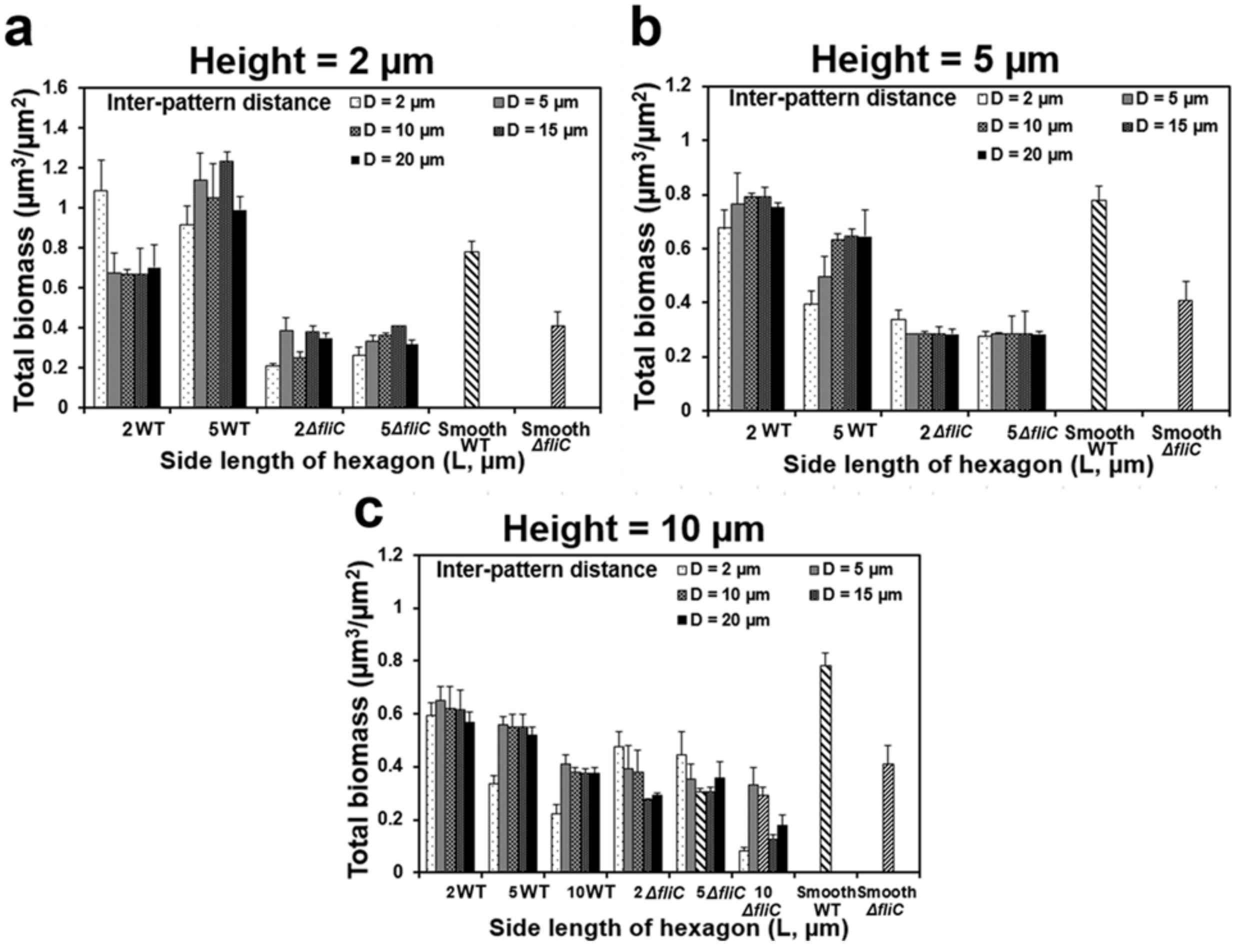

Figure S12. Biofilm formation of the WT *E. coli* and its isogenic *fliC* mutant on PDMS surfaces modified with hexagon shaped topographic patters (mean ± standard error shown; at least three biological replicates were tested for each sample). The graphs show the biomass of biofilms on surfaces modified with 2 (**a**), 5 (**b**), or 10 (**c**) µm tall hexagon shaped patterns with varied side length (2, 5, or 10 µm) and inter-pattern distance (2, 5, 10, 15, or 20 µm).

Movie S1. Rotation of a WT *E. coli* cell tethered on top of a 20 μm wide protruding line. The cell shape (circled in red) was automatically detected by ACTIVE. The movie shows that the center of cell mass followed a regular circle during rotation (based on images collected over 198 s). A few other cells were also seen with short presence during the movie. These are planktonic cells and not the focus of this study.

Movie S2. Rotation of WT *E. coli* cells on the edge of a narrow (5 μm wide) protruding line. The movie shows that the cell body moved in an irregular pattern (based on images collected over 41 s).

References:

1 Parkinson, J. S. & Houts, S. E. Isolation and behavior of *Escherichia coli* deletion mutants lacking chemotaxis functions. *J. Bacteriol.* **151**, 106-113 (1982).

2 Blair, D. F., Kim, D. Y. & Berg, H. C. Mutant MotB proteins in *Escherichia coli*. *J. Bacteriol.* **173**, 4049-4055 (1991).

3 Diao, J. P., *et al*. A Three-channel microfluidic device for generating static linear gradients and its application to the quantitative analysis of bacterial chemotaxis. *Lab Chip* **6**, 381-388 (2006).

4 Park, S. *et al.* Influence of topology on bacterial social interaction. *Proc. Natl. Acad. Sci. USA* **100**, 13910-13915, (2003).

5 Rahme, L. G. *et al.* Common virulence factors for bacterial pathogenicity in plants and animals. *Science* **268**, 1899-1902, (1995).

6 Choi, J. Y. *et al.* Identification of virulence genes in a pathogenic strain of *Pseudomonas aeruginosa* by representational difference analysis. *J. Bacteriol.* **184**, 952-961 (2002).

7 Liberati, N. T., *et al.* An ordered, nonredundant library of *Pseudomonas aeruginosa* strain PA14 transposon insertion mutants. *Proc. Natl. Acad. Sci. USA* **103**, 19931-19931, (2006).

8 Hou, S., Gu, H., Smith, C. & Ren, D. Microtopographic patterns affect *Escherichia coli* biofilm formation on poly(dimethylsiloxane) surfaces. *Langmuir* **27**, 2686-91 (2011).
